# Synergistic Enhancement of Li-O_2_ Battery Capacity and Cycle Life Using Carbon Nanochain/Multiwall Carbon Nanotube Composites

**DOI:** 10.3390/ma18163897

**Published:** 2025-08-20

**Authors:** Michael D. Womble, Cynthia Adebayo, Silas Cascio, Michael J. Wagner

**Affiliations:** Department of Chemistry, The George Washington University, Washington, DC 20052, USA

**Keywords:** biochar, valorization, nanomaterial, laser pyrolysis, energy storage

## Abstract

Multiwalled carbon nanotube (MWCNT) Li-O_2_ cathodes can achieve high gravimetric capacity. However, the macropores of these cathodes require a relatively large mass of electrolyte to fill, resulting in lower true gravimetric and volumetric capacities. Here we report a simple method to incorporate a mesoporous material, carbon nanochains (CNCs), into the macropores of MWCNTs, resulting in composite cathodes that fully utilize their pore structure to store Li_2_O_2_ product. The composite cathodes exhibit additional mesopores with an average diameter of ~100 nm. This results in dramatic increases in true gravimetric (21% to 870 mAh/g) and volumetric (>200% to 5664 mAh/cm^3^) capacities. The composite cathodes demonstrate improved electrochemical reversibility, increasing the cycle life by more than 50%.

## 1. Introduction

Li-O_2_ battery chemistry is of great interest due to its high theoretical energy densities (~3500 Wh/kg) [1,2,3,4], an order of magnitude larger than current Li-ion batteries (250–270 Wh/kg) [5]. The use of carbon cathodes also means that there is the potential for cheaper batteries than market dominate Li-ion batteries (LIBs), currently priced at approximately USD 150/Wh [6]. Carbon nanotubes (CNTs) (both single-wall (SWCNTs) and multiwall (MWCNTs) structures) have established themselves as the most studied cathode material due to their large pore volumes, pore diameters, good electrical conductivity, and good chemical stability [7,8,9,10,11,12,13,14,15].

Previously, MWCNT cathodes were compared to those composed of a structurally similar material, carbon nanochains (CNCs) [16,17]. The former were found to possess large gravimetric capacities, averaging 5290 ± 630 mAh/g. However, due to their low cathode density (0.505 g/cm^3^), their volumetric capacity is rather low, averaging 2333 ± 256 mAh/g. SEM images of fully discharged MWCNT cathodes revealed that even after discharge they still possessed a large fraction of open pore volume, with the Li_2_O_2_ particles never exhibiting interparticle growth, remaining distinctly individual, and often relatively well distanced from each other. Thus, while MWCNTs possess good gravimetric capacity they make poor use of their pore volume to store Li_2_O_2_. CNC cathodes, like MWCNT cathodes, were found to possess large gravimetric capacities (4870 ± 450 mAh/g) but much larger volumetric capacities (5113 ± 580 mAh/g), due in part to their higher measured density (1.055 g/cm^3^). Importantly, Li_2_O_2_ toroidal particles form a dense film of interconnected, rather than isolated, particles on the CNC cathodes. The higher volumetric capacity and formation of dense discharge films yielded a significantly larger true gravimetric capacity [18] (845 ± 13 and 717 ± 42 mAh/g, respectively).

In this work, CNCs and MWCNTs were mixed together to make CNC/MWCNT composite cathodes at various mass fractions, filling the unutilized pore volume in the MWCNT cathodes with CNCs to improve their volumetric capacity. In addition, CNCs possess larger surface areas than MWCNTs (205.9 m^2^/g and 93.7, respectively), potentially giving the composite a high number of O_2_ reduction sites, while MWCNTs provide structural integrity and better electrical conductivity. It has been suggested that discharge in MWCNT cathodes is limited by surface passivation [19,20,21], stopping the formation of Li_2_O_2_ before the full pore volume of the MWCNT cathode is fully utilized. This might be overcome by increasing the number of available reaction sites present in the cathode, allowing for the formation of Li_2_O_2_ to progress further, resulting in larger discharge capacities. Finally, the addition of a primarily mesoporous carbon, CNCs, into the large macropores of MWCNT cathodes allows for the investigation of cell performance as a function of the density of the most Li-O_2_ active (>100 nm diameter) pores and cathode macroporosity. Two previous studies suggest that a bimodal combination of meso- and macropores can improve discharge capacity [22,23]. Here we show that the full depth of discharge capacities, both gravimetric and volumetric, and cycling lifetimes are highly dependent on the CNC/MWCNT cathode composition and the consequent pore structure, exhibiting performance that is significantly better than the cathodes of either pure material.

## 2. Experimental Section

All data points presented are an average of measurements made with three or more samples.

### 2.1. Materials

All materials were used as received unless stated otherwise. Cellulose (FMC BioPolymer, Philadelphia, PA, USA) was used as the biomass starting material. Iron (II) chloride tetrahydrate, used as the catalyst, was purchased from STREM (Newburyport, MA, USA, 99%, product no. 93-2632). Nitric acid purchased from Millipore Sigma (Burlington, MA, USA, GR ACS, product no. NX0409-2) was used for CNC purification. Isopropyl alcohol (IPA) used to create CNC and MWCNT suspensions was purchased from VWR Chemicals (Radnor, PA, USA, general lab use, product no. BDH113-19L). LiNO_3_ from Alfa Aesar (Ward Hill, MA, USA, 99.98% metal basis, product no. 10985) and dimethyl sulfoxide (DMSO) from Thermo Fisher Scientific (Pittsburgh, PA, USA, 99.9%, product no. D139-1) were used to make the electrolyte. Multiwalled carbon nanotubes (>95wt%, SKU # 030103), 10–20 nm in diameter and 10–30 µm in length, were purchased from Cheaptubes.com (Grafton, VT, USA). Porous Carbon Foam (BCGDL1400S) and coin cells (CR2016 and CR2032) were purchased from MTI Inc. (Richmond, CA, USA). Whatman glass microfiber filters (Grade GF/D, CAT no. 1823-150) were purchased from Millipore Sigma.

### 2.2. CNC Preparation

CNCs were synthesized as previously reported [16]. Briefly, pellets of cellulose impregnated with 1 w% FeCl_2_·4H_2_O were heated to 1000 °C for 20 min. Following cooling, CNCs were purified by microwave digestion (PreeKem, Shanghai, China, CN, WX-6000) in nitric acid (10 mg/mL) for 120 min at 180 °C. The CNCs were isolated by vacuum filtration, rinsed with distilled water, and dried overnight at 120 °C.

### 2.3. CNC/MWCNT Cathode Preparation

CNC and MWCNTs were sonicated in IPA for 30 min to form a suspension with a concentration of 0.33 mg/mL. The suspension was then poured through carbon fiber paper (CFP), from which carbon black had been removed, stacked on top of a PTFE membrane filter (47 mm diameter, 0.45 µm pore size, Simsii Inc., Issaquah, WA, USA), leaving a layer of CNC and/or MWCNTs on top of the CFP. The sample was then dried at 120 °C overnight. Electrodes, 1.6 cm in diameter, were punched out of the film (MSK-T-07 Precision Disc Cutter, MTI Inc.) and pressed between two sheets of aluminum foil at 6000 lbs (19,400 PSI). The loading of the CNC and/or MWCNT cathodes was ~2 mg/cm^2^.

### 2.4. Characterization

Scanning electron microscopy (SEM) images of pristine and discharged cathodes were obtained using an FEI Teneo LV FEG (Thermo Fisher Scientific). Discharged cathodes were prepared by disassembling Li-O_2_ cells under an argon atmosphere inside a glovebox. Cathodes were cleaned by rinsing in DMSO and then dried under vacuum.

### 2.5. Electrochemical Cell Assembly and Testing

Coin cells (CR2016, MTI Inc.) were modified by punching nine (1 mm diameter) holes into the cathode side to allow for oxygen flow. Cells were assembled in an Ar-filled glovebox using lithium metal disk (99.9%, 15.6 mm dia., 0.45 mm thick, MTI Inc.) anodes, a microfiber glass filter (Grade GF/D, Whatman Inc., Little Chalfont, UK) separator, and 200 µL of 0.1 molar LiNO3 (99.98%, Alfa Aesar) in dimethyl sulfoxide (DMSO, 99.9%, Fisher Sci.) electrolyte.

Assembled Li-O_2_ cells were placed in air-tight custom-built PVC containers, purged with a flow of O_2_ (99.995%, Roberts Oxygen Company, Cockeysville, MD, USA) for 10 min, and then maintained at 5 psi. The cells were held for 4 h at OCV and then cycled at a current density of 0.2 mA/cm^2^. The assembled CR2016 cells were discharged from open circuit voltage (OCV) (~2.8–3.0 V) to 2.0 V to measure the maximum capacity that the CNC and MWCNT cathodes could achieve. Following the practice of previous studies [14,24,25], multicycle testing was performed with a limited depth of discharge (500 mAh/g discharge/charge) to avoid dendrite formation with CNC and MWCNT cathodes. CR2032 cells were used in multicycle testing. A bubbler filled with DMSO was used to saturate the O_2_ atmosphere inside the cycling container with DMSO to reduce the rate of electrolyte evaporation.

## 3. Results and Discussion

### 3.1. MWCNT Cathodes

As previously reported [16], MWNCT cathodes use a limited portion of their pore volume for Li_2_O_2_ storage in fully discharged cathodes. The fraction of the MWCNT cathode pore volume occupied by Li_2_O_2_ after fully discharging the cathode can be calculated as follows:

(1) $$\mathrm{MWCNTPoreVolume}\;=\;\frac{m}{\rho_{\mathrm{cathode}}}\;-\;\frac{m}{\rho_{\mathrm{true}}}$$

**Figure 1:**
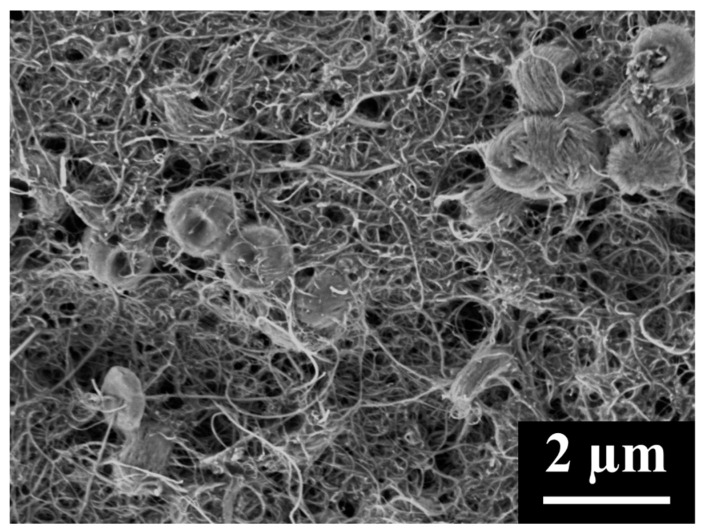
SEM image of the surface of a fully discharged MWCNT cathode.

**Figure 2:**
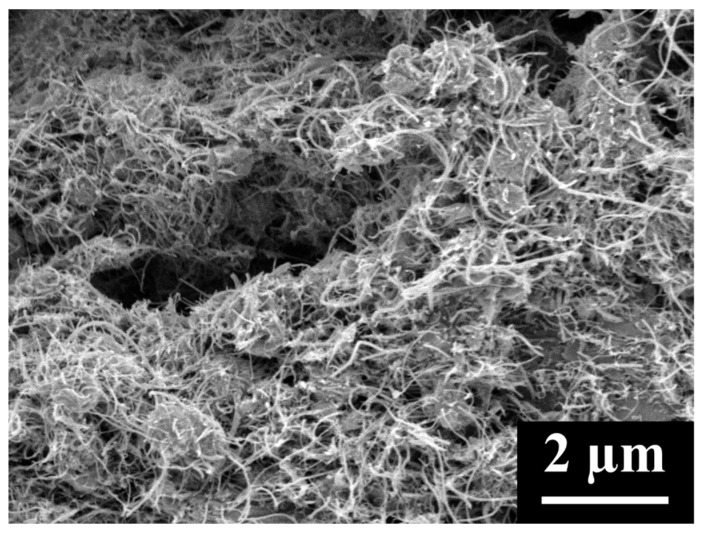
Cross-section of a fully discharged MWCNT cathode.

(2) Li2O2 Pore Volume =Q Coulombs ∗ 6.24×1018e−Coulomb∗MW2 e−Li2O2∗6×1023 Li2O2mole∗ρLi2O2

(3) $$\%\mathrm{PoreVolumeUtilized}\;=\frac{{\mathrm{Li}}_{2}O_{2}\mathrm{PoreVolume}}{\mathrm{MWCNTPoreVolume}}\times 100$$

where m is the mass of MWCNTs, $\rho_{\mathrm{cathode}}$ is the cathode density of MWCNTs (0.505 g/cm^3^), $\rho_{\mathrm{true}}$ is the true density of MWCNTs (2.1 g/cm^3^), Q is the charge the MWCNT cathode stores, MW is the molecular weight of Li_2_O_2_, and $\rho_{{\mathrm{Li}}_{2}O_{2}}$ is the density of Li_2_O_2_. Using these three equations, the average percent of pore volume utilized in MWCNT cathodes to store Li_2_O_2_ was calculated to be 97%. However, this is clearly not the case as SEM images of fully discharged MWCNT cathodes (Figure 1 and Figure 2) show a high degree of porosity, including a myriad of small pores surrounding the product particles and, in addition, the presence of very large pores. The presence of significant porosity indicates that product formation causes cathode swelling during discharge, which is not accounted for in our calculations. Density measurements of fully discharged cathodes could potentially allow for better calculations; unfortunately, they are unreliable due to the reactivity of the product and difficulty in ensuring the removal of all of the electrolyte. Thus, although it is clear that the discharged MWCNT cathodes are highly porous, the degree of porosity is uncertain.

### 3.2. CNC/MWCNT Cathode Pore Structure

**Figure 3:**
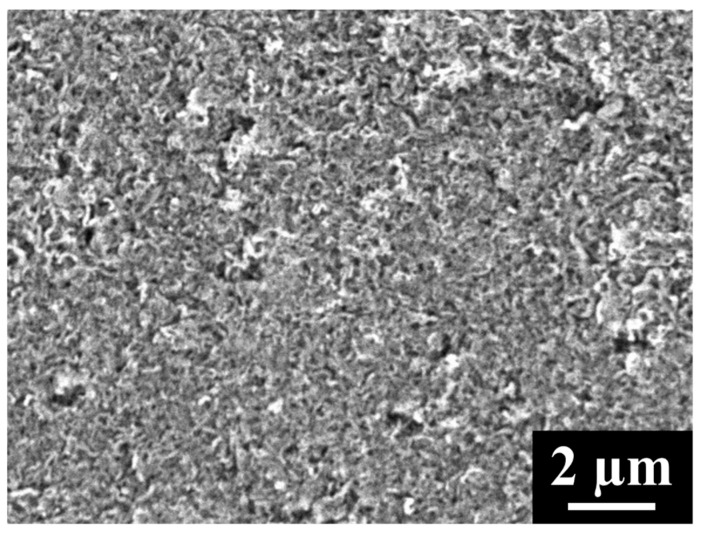
SEM image of pristine CNC cathode.

**Figure 4:**
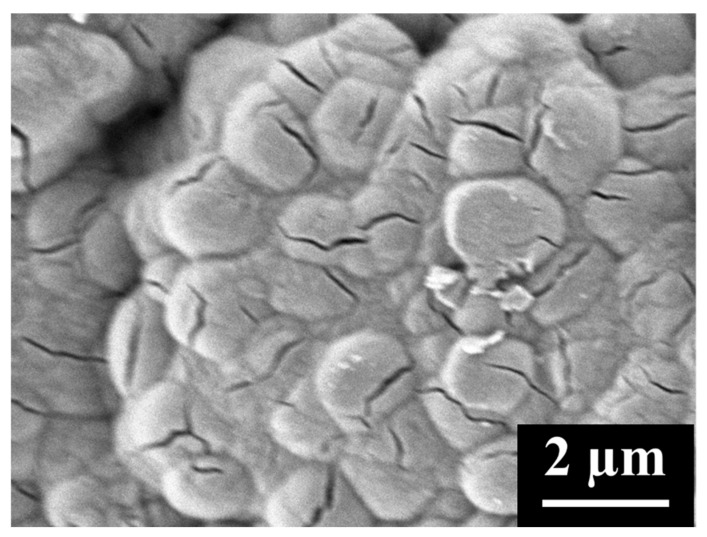
SEM image of the surface of a fully discharged CNC cathode.

Compared to MWCNTs, CNCs are a much denser carbon material with smaller pores that appear to remain unblocked even after the cathode is fully discharged [16]. CNC and MWCNT cathodes both possess similar pore volumes from pores that are 100 nm in diameter or less (0.68 cm^3^/g for CNCs and 0.60 cm^3^/g for MWCNTs), a size range found to be the most effective for Li-O_2_ battery performance [19]. CNC cathodes, unlike those of MWCNTs, possess very few pores larger than 100 nm in diameter, pores that are thought to be too large to contribute significantly to Li-O_2_ capacity (Figure 3). Rather than storing Li_2_O_2_ in pores, CNC cathodes store it on the surface (Figure 4). A composite cathode can be created by mixing CNCs into MWCNTs, in which the CNCs fill in much of the unused pore volume in the MWCNTs. This increases the number of pores that are 100 nm or less in diameter, a size range that is believed to be important for the continuous diffusion of oxygen through the cathode during discharge [19,23], providing the composite cathode with additional reaction sites. The optimal filling of the MWCNT macropores with CNCs is that which maximizes the density of those active pores while maintaining sufficient macroporosity for Li_2_O_2_ storage.

**Figure 5:**
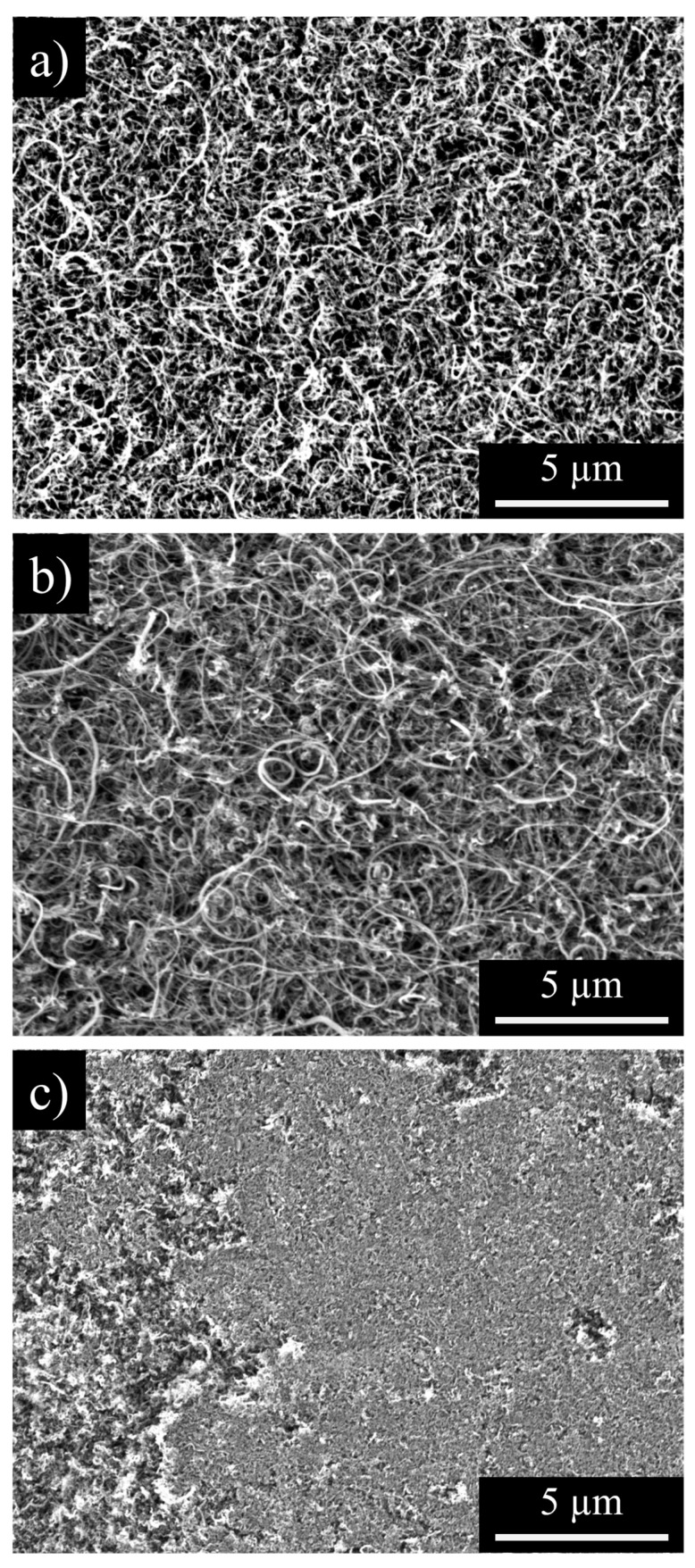
SEM of pristine (**a**) MWCNT, (**b**) 50CNC/50MWCNT, and (**c**) CNC cathodes.

The changing pore structure and morphology of the CNC/MWCNT cathodes as a function of CNC mass fraction can be seen in SEM images (Figure 5). Pristine MWCNT cathodes (Figure 5a) show a highly porous structure with pores that are approximately 1 µm in diameter created by the entanglement of the MWCNTs. As CNCs are mixed into the MWCNTs (Figure 5b, 50% of each by mass), they fill in the larger pores in the MWCNTs, decreasing the porosity and increasing the density of the cathode. When the cathode is entirely composed of CNCs (Figure 5c), pores larger than ~100 nm in diameter are almost completely gone, resulting in a highly dense and relatively uniform surface.

**Figure 6:**
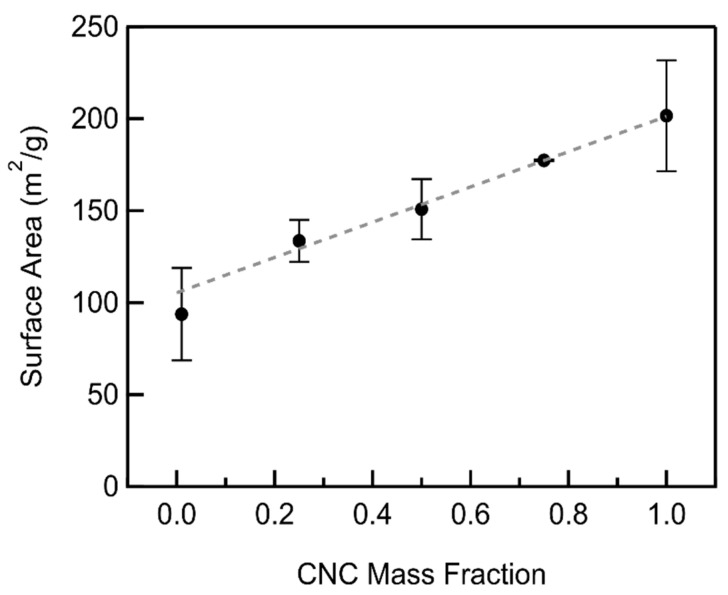
Surface area of CNC/MWCNT cathodes as a function of CNC mass fraction. The weighted linear least-squares fit to the rule of mixtures is shown as a dashed line.

The CNC/MWCNT cathode surface area was found to increase linearly with increasing the CNC mass fraction (Figure 6, Table S1). CNCs possess larger surfaces areas (205.9 m^2^/g) than MWCNTs (93.7 m^2^/g). Therefore, as the CNC mass fraction increases, the total surface area of the composite cathodes increases linearly in agreement with the rule of mixtures [20].

(4) $$SSA_{composite}=f\times SSA_{CNCs}+\left(1-f\right)\times SSA_{MWCNTs}$$

where $SSA_{composite}$ is the specific surface area of the CNC/MWCNT composite, and f is the CNC mass fraction. A linear least-squares fit of the CNC/MWCNT surface area as a function of mass fraction, weighted by the standard deviations, returns surface areas of 105.4 ± 13.4 m^2^/g and 201.3 ± 4.5 m^2^/g for MWCNTs and CNCs, respectively.

**Figure 7:**
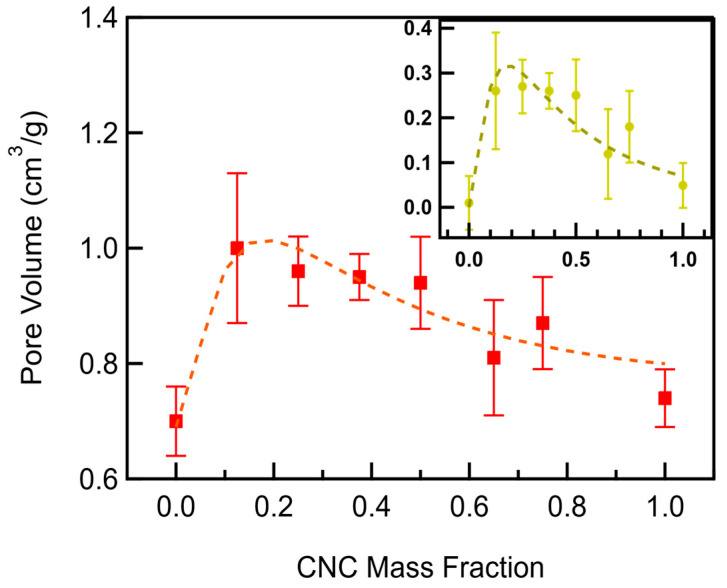
Pore volume of CNC/MWCNT cathodes as a function of CNC mass fraction. The weighted log-normal plus line least-squares fit is shown as a red dashed line. The log-normal least-squares fit to the additional porosity is shown in the inset.

The pore volume of CNC/MWCNT cathodes increases with an increasing CNC mass fraction up to 0.2 and decreases at greater CNC mass fractions (Figure 7, Table S1). The vast majority of the macroporosity of MWCNTs consists of pores that are too large to be measured by nitrogen gas adsorption isotherms, in excess of 100 nm in diameter. Adding CNCs to MWCNTs increases the measured porosity because they occupy unmeasured macropores while providing additional mesoporosity. Based on the rule of mixtures, a linear increase in mesoporosity is expected, but clearly not observed. Instead, a sharp increase in mesoporosity occurs as the CNC mass fraction increases to 0.2 before decreasing more steadily as the CNC mass fraction approaches 1. The increase in mesoporosity in CNC/MWCNT cathodes beyond that of either of the constituents indicates that additional mesopores are formed, likely at contact points between the CNCs and the MWCNTs. The random manner in which contact points between CNCs and MWCNTs occur and the physical impossibility of negative pore volume combined with the shape of the peak indicate a log-normal distribution of pore volume as a function of the CNC mass fraction. Thus, the measured porosity was fit to the combination of the linear contribution of the rule of mixtures plus the log-normal contribution of the additional porosity given by

(5) $$f\left(x\right)=b+mx+\frac{A}{x\sigma \sqrt{2\pi }}e^{\left(\frac{-{\left(ln\left(x\right)-\mu \right)}^{2}}{2\sigma^{2}}\right)}$$

where b is the y-intercept and m the slope of the linear porosity contributions of the MWCNTs and CNCs, A is the peak amplitude of the log-normal contribution of the additional porosity, x is the CNC mass fraction, µ is the mean of the CNC mass fraction’s natural logarithm, and σ is the standard deviation of the CNC mass fraction’s natural logarithm. The results of the fits are shown in Figure 7, and the values determined for the parameters are listed in Table S2.

The CNC mass fraction at which the most additional mesoporosity occurs is approximately 0.2. When the CNC mass fraction is greater than 0.2, CNCs increasingly contact each other rather than making additional contacts, and, consequently, fewer mesopores are created between CNCs and MWCNTs. As the mass fraction increases further, the number of mesopores forming between CNCs and MWCNTs decreases until the mesoporosity of a pure CNC cathode is reached.

**Figure 8:**
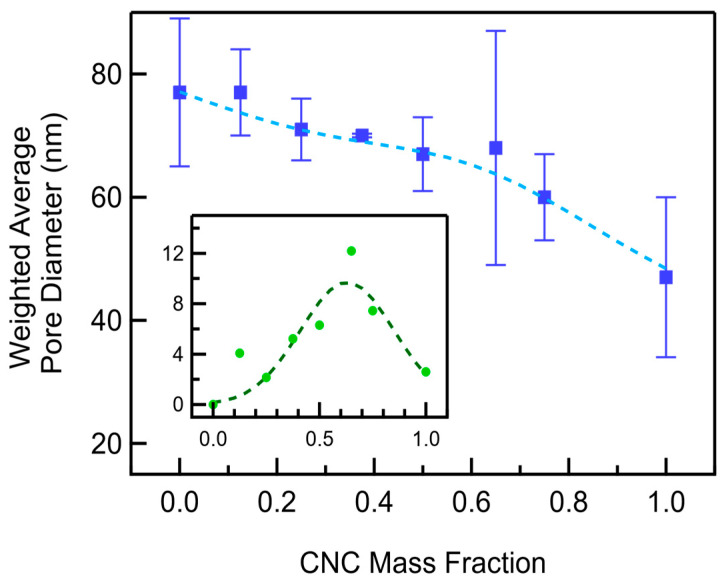
Weighted average pore diameter of CNC/MWCNT cathodes as a function of CNC mass fraction. The weighted log-normal plus line least-squares fit is shown as a dashed line. The Gaussian least-squares fit to the additional pore diameter beyond that predicted by the rule of mixtures is shown in the inset as a dashed dark green line.

The CNC/MWCNT weighted average pore diameter decreases as a function of an increasing CNC mass fraction (Figure 8, Table S1). CNC/MWCNT cathodes with a 0.5 CNC mass fraction showed a 13% decrease in their weighted average pore diameters compared to MWCNT cathodes, decreasing from an average of 77 nm to 67 nm. As the CNC mass fraction increases from 0.5 to 1, a 30% decrease in pore diameter is seen, from 67 nm to 47 nm. This deviation from that linearity predicted by the rule of mixtures occurs because the size of the additional mesopores created between CNC and MWCNTs upon the addition of CNCs is larger than the mesopores of the CNCs. The addition of larger mesopores increases the weighted average pore diameter in CNC/MWCNT cathodes relative to what is predicted by the rule of mixtures, resulting in a negatively skewed distribution that can be reasonably fit by a Gaussian plus linear function (Equation (6)).

(6) $$f\left(x\right)=\;b+\mathrm{mx}+\frac{A}{\sigma \sqrt{2\pi }}e^{\left(-\frac{{\left(\ln\left(x\right)-\mu \right)}^{2}}{2\sigma^{2}}\right)}$$

where b is the y-intercept of baseline, m is slope of baseline, A is the peak amplitude, x is the CNC mass fraction, µ is the mean, and σ is the standard deviation. Fit parameter values are listed in Table S2. The additional pores formed at a low CNC mass fraction are larger than those at a high mass fraction, peaking at a mass fraction of ~0.63, given by µ. The peak position occurs at a much higher mass fraction than that observed in the total porosity (Figure 7), indicating that while the size of the pores formed increases, their gravimetric number density decreases. This is consistent with a decrease in the number of contact points between CNCs and MWCNTs with a decreasing MWCNT mass fraction, as expected.

**Figure 9:**
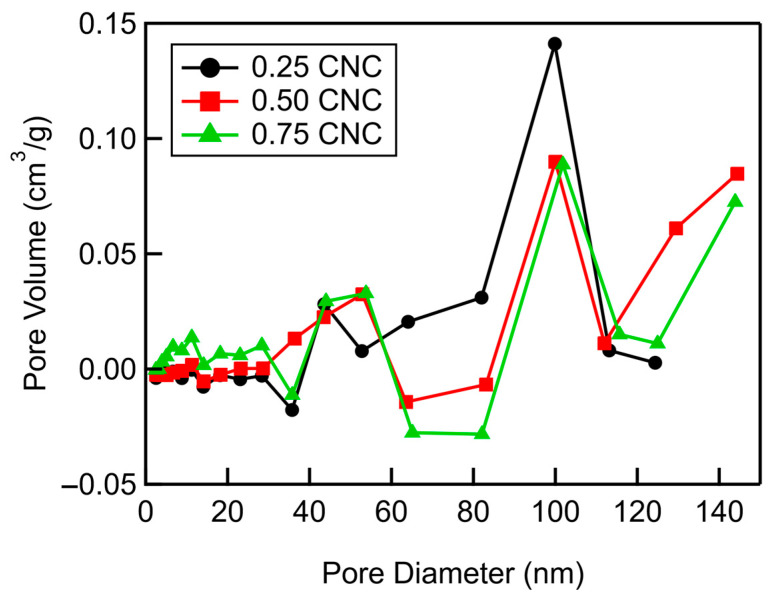
Pore size distributions of additional mesopores in CNC/MWCNT cathodes. The data points are connected by lines to guide the eye.

The pore size distribution of the additional mesopores created by mixing CNCs into MWCNTs shows that most of the additional mesopores are around 100 nm in diameter across all CNC mass fractions (Figure 9). These pore size distributions of the additional mesopores in CNC/MWCNT cathodes were determined by subtracting the predicted pore size distributions of CNC/MWCNTs cathodes (calculated using the rule of mixtures) from the pore size distributions measured from gas adsorption isotherms. At a CNC mass fraction of 0.25, additional mesopores with diameters between 40 and 80 nm are also present. As the CNC mass fraction increases to 0.5 and then 0.75, the number of additional pores ranging from less than ~80 nm and larger than 100 nm decreases and increases, respectively, increasing the weighted average pore diameter with a maximum value at ~0.63, as seen in the inset in Figure 8.

### 3.3. CNC/MWCNT Cathode Performance

**Figure 10:**
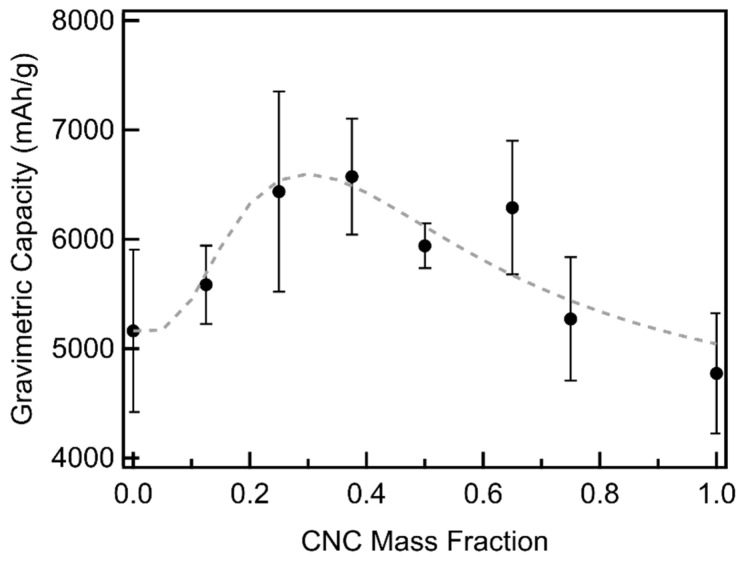
Full depth of discharge gravimetric capacity of CNC/MWCNT cathodes as a function of CNC mass fraction. The weighted least-squares log-normal fit is shown as a dashed line.

CNC/MWCNT composite cathodes obtain larger full depth of discharge gravimetric capacities than those achieved by either pure MWCNTs or CNCs (Figure 10, Table S1), peaking at a CNC mass fraction of 0.375 with an average of ~6560 mAh/g. Some cells reached capacities as large as 7300 mAh/g, a 47% increase in gravimetric capacity compared to pure CNCs and a 37% increase in gravimetric capacity compared to pure MWCNTs (note, our finding are within the range of capacity values for pure MWCNTs found in previous studies) [8,9,10,11,12,13,14,15]. The measured gravimetric capacities follow a log-normal distribution as a function of CNC mass fraction and were fit using the log-normal plus a linear function shown in Equation (5) (dashed line in Figure 10) with fit parameter values listed in Table S2. The CNC mass fraction of the maximum gravimetric capacity obtained from the fit, 0.33, is only slightly larger than that found for the maximum pore volume (Figure 7) and near that found for the average pore diameter (Figure 8).

**Figure 11:**
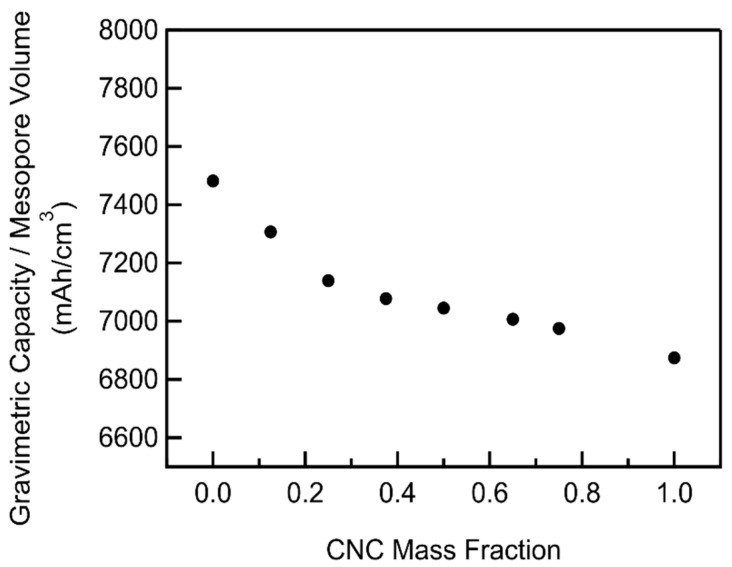
Full depth of gravimetric capacity to mesopore volume ratio as a function of CNC mass fraction calculated from the log-normal fits of CNC/MWCNT mesopore volume and gravimetric capacities.

The functional form of the dependence of the gravimetric capacity on mass fraction is remarkably similar to that of the total additional mesoporosity, indicating their close relationship. Additionally, the gravimetric capacity decreases as a function of mesopore volume with increasing CNC mass fractions (Figure 11), corresponding to a decrease in average pore diameters (Figure 8). This indicates that while gravimetric capacity is largely determined by total mesopore volume, larger pores result in a higher capacity than smaller ones, given the equivalent mesopore volumes. Smaller pores confine the reaction intermediates, such as O_2_^−^, closer to the cathode. This in turn increases the rate of parasitic reactions that coat and passivate the carbon cathode with Li_2_CO_3_, decreasing the capacity [21].

**Figure 12:**
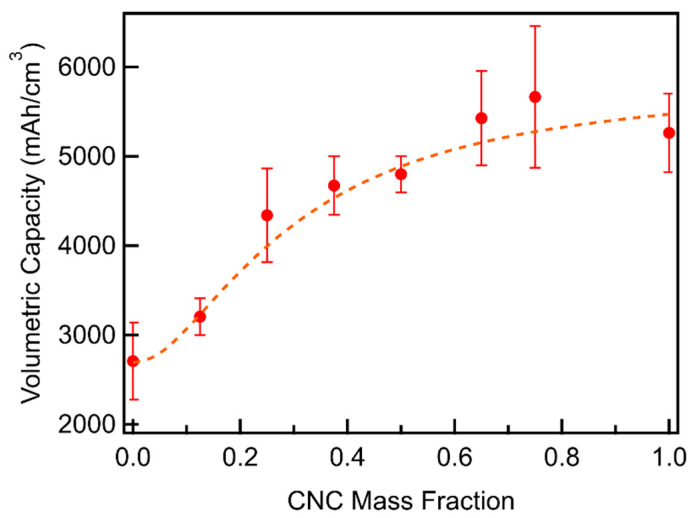
Full depth of discharge volumetric capacity of CNC/MWCNT cathodes as a function of CNC mass fraction. The weighted log-normal times line least-squares fit is shown as a dashed line.

Volumetric capacities (Figure 12, Table S1) show a steady increase up to a mass fraction of 0.65 with little subsequent increase. This behavior can be understood by looking at the relationship between volumetric capacity, gravimetric capacity, and density given as

(7) $$Volumetric\;Capacity\left(\frac{mAh}{cm^{3}}\right)=Gravimetric\;Capacity\left(\frac{mAh}{g}\right)\times Density\left(\frac{g}{cm^{3}}\right)$$

**Figure 13:**
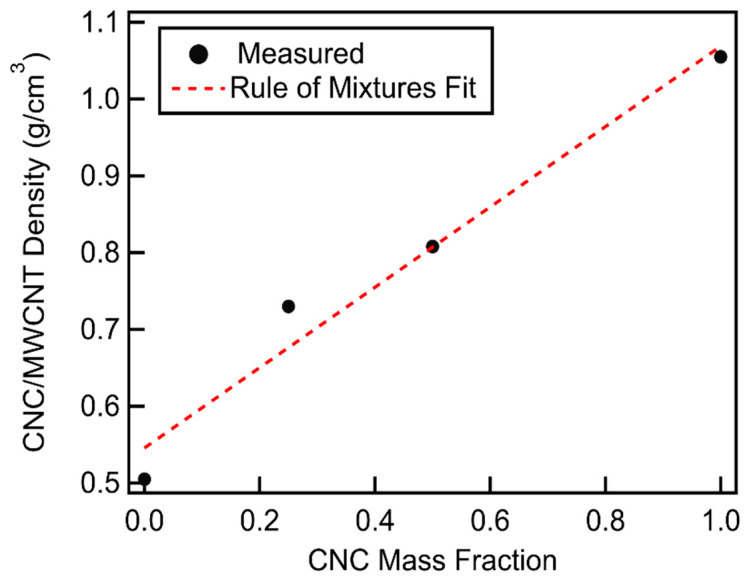
Densities of CNC/MWCNT cathodes as function of CNC mass fraction. The fit to rule of mixtures is shown as a dashed line.

As the CNC mass fraction increases, the CNCs fill in the unused pore volume in the MWCNT cathodes, creating a denser cathode. The density of the CNC/MWCNT cathodes increases linearly with the CNC mass fraction in accordance with the rule of mixtures given in Equation (5) and shown in Figure 13. Multiplying the gravimetric capacity, displaying a log-normal distribution, with the CNC/MCWNT density, which is linearly increasing with the CNC mass fraction, results in volumetric capacities that increase monotonically. Thus, the volumetric capacity is best fit by multiplying a log-normal function by a linear function and adding a linear function, shown by the dashed line in Figure 12, and given by the function given in Equation (8). Fit parameter values are listed in Table S2.

(8) $$f\left(x\right)=\;b+\mathrm{mx}+\left(\frac{A}{\sigma \sqrt{2\pi }}\right)e^{\left(-\frac{{\left(\ln\left(x\right)-\mu \right)}^{2}}{2\sigma^{2}}\right)}+\frac{A}{x\sigma \sqrt{2\pi }}e^{\left(-\frac{{\left(\ln\left(x\right)-\mu \right)}^{2}}{2\sigma^{2}}\right)}$$

**Figure 14:**
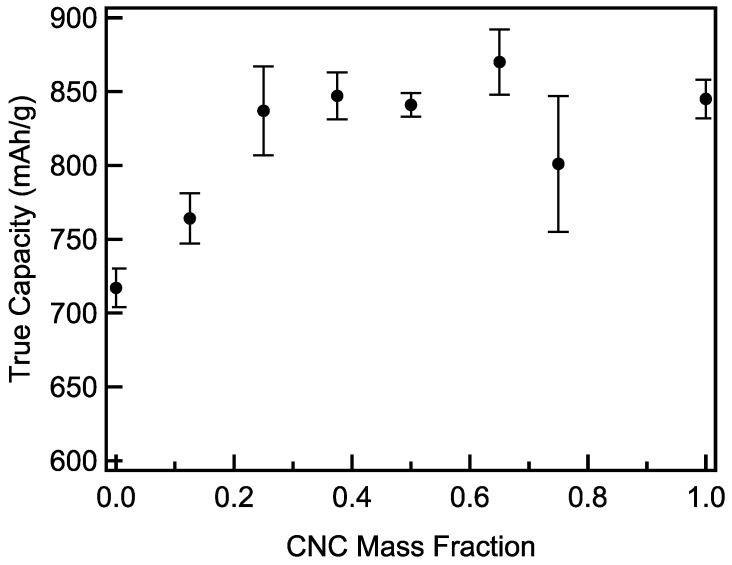
Full depth of discharge true capacities of CNC/MWCNT cathodes as a function of CNC mass fraction.

Gravimetric capacities based only on the mass of the storage material can greatly distort the capabilities of Li-O_2_ cathodes by neglecting the mass of the electrolyte that fills their pores and that of the Li_2_O_2_ product. Thus, comparisons of Li-O_2_ cathodes to each other or to cathodes of other battery chemistries (e.g., Li-ion) based on simple gravimetric capacities are generally invalid. True capacities [18], which take the mass of the electrolyte in the cathode pores and Li_2_O_2_ product into account, allow for valid comparisons. Here they show a steady increase with an increasing CNC mass fraction up to a CNC mass fraction of 0.25 with little or no increase at higher fractions (Figure 14, Table S1). MWCNTs possess large macropore volumes that require electrolyte to fill, and thus relatively low true capacities. As the CNC mass fraction increases, filling in the macropores, the contribution of the mass of electrolyte diminishes. At higher mass fractions, the decreasing gravimetric capacity is offset by further decreases in the pore volume, resulting in a relatively unchanged true capacity.

**Figure 15:**
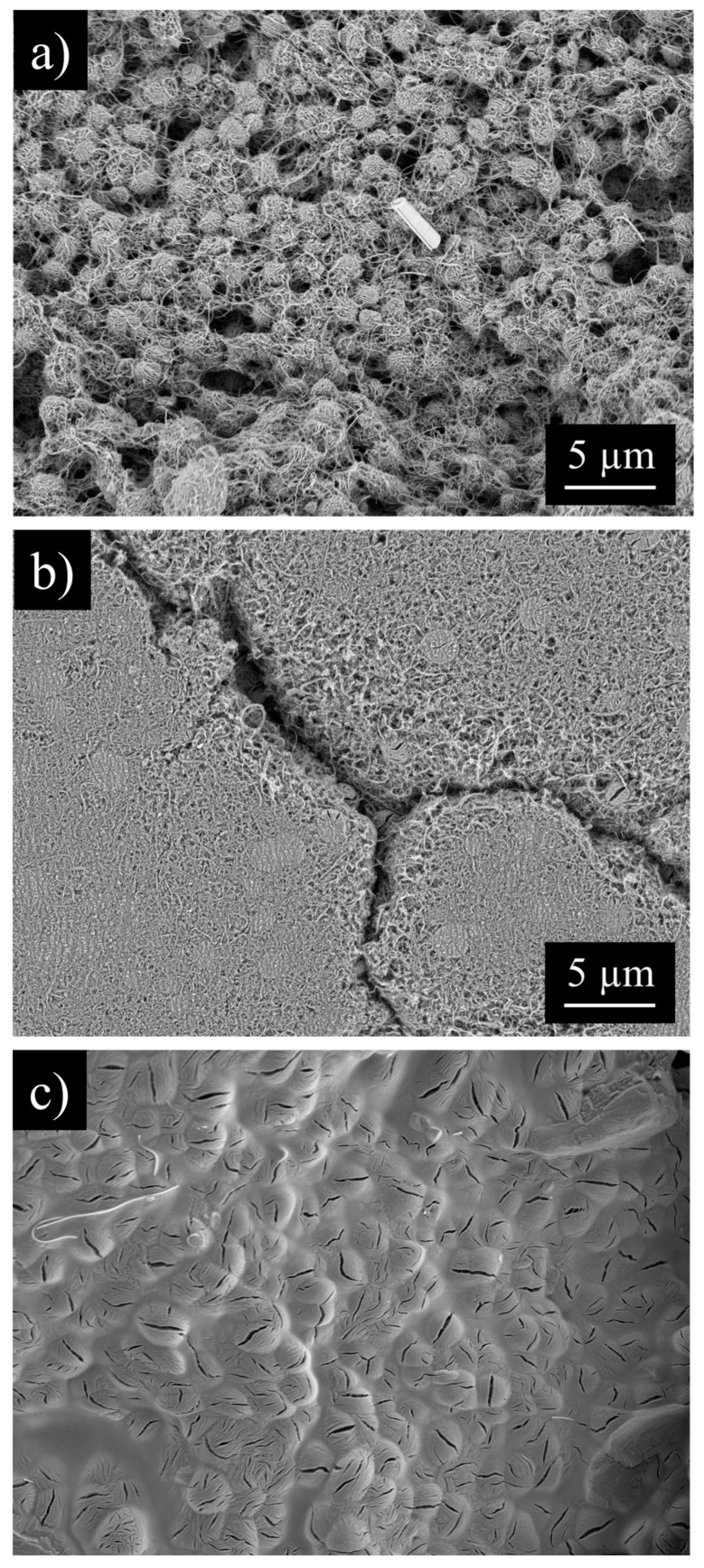
SEM images of discharged (**a**) MWCNT, (**b**) 50/50 CNC/MWCNT, and (**c**) CNC cathodes.

Li_2_O_2_ toroidal particles form inside pure MWCNT cathodes (Figure 15a), entangled in the MWCNTs. The discharged MWCNT cathode shows many large pores within which Li_2_O_2_ has not formed; the MWCNT cathode does not fully utilize its pore volume during discharge. CNC/MWCNT cathodes at a CNC mass fraction of 0.5 retain mesoporosity, but Li_2_O_2_ appears to fully occupy nearly all macropores, having formed almost entirely within the cathode (Figure 15b). In addition, cracks form in which Li_2_O_2_ particles can be seen, suggesting that the cathode expanded. In pure CNC cathodes, Li_2_O_2_ particles completely cover the surface in a thick film in which the toroidal Li_2_O_2_ particles have intergrown.

Cathode porosities as a function of CNC mass fraction are given in Table S3, as calculated using

(9) $$\%\;Porosity=\frac{PV_{cathode}-\left(\frac{m}{\rho_{True}}\right)}{PV_{cathode}}\times 100$$

(10) $$PV_{macro}=PV_{cathode}-PV_{meso}$$

where PV_cathode_ is the pore volume of the cathode (Equation (1)), m is the combined mass of MWCNTs and CNCs, and ρ_true_ is the true density of the cathode (2.1 g/cm^3^). PV_meso_ is the combined mesopore volume of CNCs and MWCNTs and calculated using the log-normal fit shown in Figure 7. PV_macro_ is the combined macropore volume of CNC and MWCNTs obtained from subtracting the calculated mesopore volume (PV_meso_) from the cathode volume (PV_cathode_). The volume of the Li_2_O_2_ product of full discharge, calculated using Equation (2), greatly exceeds the macroporosity of the cathodes at all CNC mass fractions. While it is apparent that significant, perhaps even most or all, mesoporosity is retained after full discharge, its inclusion is not sufficient to account for the Li_2_O_2_ volume which exceeds total porosity for all formulations other than pure MWCNTs. The increase in capacity with the addition of CNCs is accompanied by a decrease in macroporosity, resulting in a product volume of greater than 500% of the macroporosity of cathodes with CNC mass fractions exceeding 0.375, the formulation with the largest observed capacity. Thus, it is clear that the growth of peroxide within the macropores causes cathode expansion; however, expansion greater than ~500% results in electrode cracking and surface deposition and a further decrease in the capacity as a function of mesoporosity.

**Figure 16:**
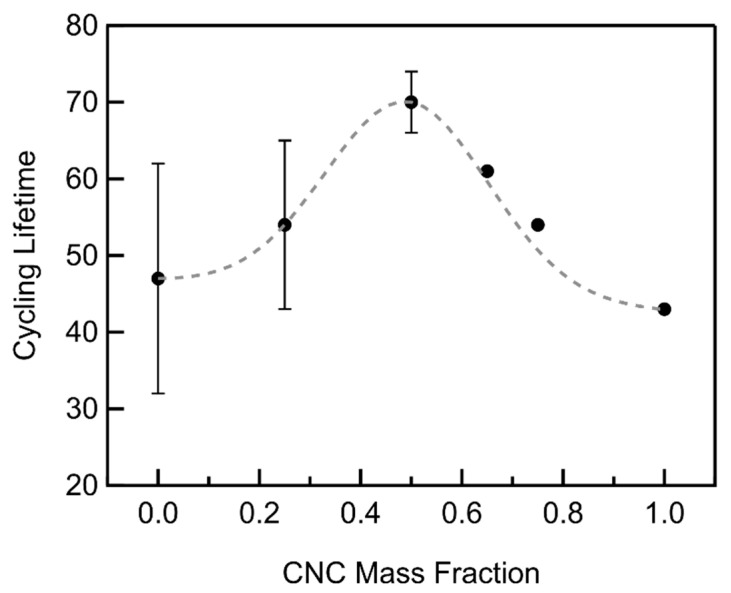
Cycling lifetimes of CNC/MWCNT cathodes as a function of CNC mass fraction. The weighted Gaussian least-squares fit is shown as a dashed line.

Cycling lifetimes follow a Gaussian distribution with the CNC mass fraction displaying maximum cycling lifetimes at a mass fraction of 0.5 where an average of 71 cycles were achieved (Figure 16, Table S1), a 51% and 65% increase over that of pure MWCNT and CNC cathodes, respectively. The cycle life was fit using Equation (11):

(11) $$f\left(x\right)\;=\;b+\frac{A}{\sigma \sqrt{2\pi }}e^{\left(-\frac{{\left(\ln\left(x\right)-\mu \right)}^{2}}{2\sigma^{2}}\right)}$$

where b is the baseline, A is the peak amplitude, x is the CNC mass fraction, µ is the mean, and σ is the standard deviation.

The pore structure plays a critical role not only in the gravimetric capacity but in the cycle life as well. The cycle life is enhanced for all composite compositions, implying that the additional pores display better reversibility. Furthermore, its functional dependence is the same as that of additional pore diameter (Figure 8, inset), peaking at similar mass fractions, suggesting that larger mesopores are responsible for the enhancement, perhaps due to reduced pore clogging.

Cycle life is optimized at a somewhat higher CNC mass fraction (0.5) than the full depth of discharge gravimetric capacity (0.375). The amount of Li_2_O_2_ that forms at this limited (500 mAh/g) depth of discharge occupies approximately 38% and 46% of the cathodes macropore volume at CNC mass fractions of 0.375 and 0.5, respectively. Therefore, cathode expansion may be avoided, with sufficient volume for product storage for either composition. The mesopore volume and average pore diameters of the samples are nearly identical; however, the longer cycling cathode has a larger surface area, implying that surface passivation is responsible for cell failure. Further increases to the mass fraction also increase surface area, but this is accompanied by a decreasing mesopore diameter and macroporosity and an increasing degree of macropore filling, indicating that pore clogging limits cycle life at high mass fractions.

## 4. Conclusions

MWCNT Li-O_2_ cathodes store Li_2_O_2_ in a fraction of their macropore volume, with the electrode swelling to accommodate the product rather than fully utilizing their porosity. Essentially, full utilization can be achieved by mixing CNCs into MWCNTs to create CNC/MWCNT composite cathodes, dramatically decreasing macroporosity while increasing mesoporosity and storage capacity. The maximum volumetric (5664 mAh/cm^3^) and true gravimetric (870 mAh/g) capacities are more than 200% and 21% greater than pure MWCNT cathodes, and approximately four times and an order of magnitude larger, respectively, than what is achievable by LIBs (150–210 mAh/g and 550–700 mAh/cm^3^) [26].

CNC occupation of the macropores between the loosely woven MWCNTs primarily creates larger-diameter (>80 nm) mesopores, presumably at the interfaces of the two materials. These larger mesopores are not only found to result in higher gravimetric capacities than smaller ones, given equivalent total mesopore volumes, but also show better electrochemical reversibility. This dramatically improves the cycle life, optimized at a CNC mass fraction of 0.5 with a 51% and 67% increase over pure MWCNT and CNC cathodes, respectively.

In summary, the composition of CNC/MWCNT composite cathodes can be optimized for lifetime, true gravimetric capacity, and volumetric capacity, although not simultaneously. CNC/MWCNT cathodes obtained their maximum cycle life at a CNC mass fraction to 0.5. The true gravimetric capacity is essentially maximized at that mass fraction, and the volumetric capacity is 77% greater than that obtained with pure MWCNT cathodes but 15% lower than its maximum value (5664 mAh/cm^3^). Further increases in the CNC mass fraction to 0.75 resulted in maximum volumetric capacity (5664 mAh/cm^3^), as the cathode density increased with the CNC mass fraction, but this resulted in a 35% decrease in the cycle life (relative to CNC mass fraction of 0.5) due to the decrease in both mesopore volume and average pore diameter.

## Data Availability

The original contributions presented in this study are included in the article/Supplementary Material. Further inquiries can be directed to the corresponding author.

## Supplementary Materials

The following supporting information can be downloaded at: https://www.mdpi.com/article/10.3390/ma18163897/s1, Table S1: Summary of the surface areas, porosities and cycling characteristics of CNC/MWCNT cathodes; Table S2: Parameters determined from data fitting detailed in the manuscript.; Table S3: Total porosity, meso- and macroporosity of CNC/MWCNT cathodes as a function of CNC mass fraction, and the volume of the Li_2_O_2_ as the result of full discharge for each formulation.
