# Supplementary material for: Synergistic Enhancement of Li-O2 Battery Capacity and Cycle Life Using Carbon Nanochain/Multiwall Carbon Nanotube Composites
Source: Materials (Basel). 2025 Aug 20;18(16):3897. doi: 10.3390/ma18163897 (PMC12387732; doi:10.3390/ma18163897)
Supplement: Supplementary file 1 [file materials-18-03897-s001.zip › materials-3797189-supplementary.pdf]

CNC mass fraction	Surface Area (m^2/g)	Pore Volume (cm^3/g)	Weighted Average Pore Diameter (nm)	Cycling Lifetimes (cycles)	Gravimetric Discharge Capacity (mAh/g)	Volumetric Discharge Capacity (mAh/g)	True Capacity (mAh/g)
0	93.7 ± 25.1	0.70 ± 0.06	77 ± 12	32 ± 3	5289 ± 769	2768.8 ± 429.5	718.2 ± 35.3
0.125	126 ± 17.8	0.95 ± 0.11	77 ± 7		5583.7 ± 357.7	3205.1 ± 205.3	763.6 ± 17.2
0.25	133.6 ± 11.4	0.96 ± 0.06	71 ± 5	65 ± 14	6435 ± 915.7	4500.0 ± 392.5	836.5 ± 29.9
0.375	145.9 ± 22.9	0.95 ± 0.04	70 ± 0.3		6200.0 ± 530.8	4673.3 ± 328.7	846.9 ± 16.0
0.5	150.9 ± 16.4	0.94 ± 0.08	67 ± 6	71 ± 4	5939.8 ± 205.5	4794.3 ± 202.6	840.7 ± 8.1
0.65	169.8 ± 21.3	0.81 ± 0.10	68 ± 19	68 ± 6	6288.8 ± 611.2	5427.2 ± 527.5	870.2 ± 21.5
0.75	177.3 ± 0.4	0.87 ± 0.08	60 ± 7	60 ± 12	5270.7 ± 565.1	5664.2 ± 792.5	801.2 ± 46.5
1	205.9 ± 26.1	0.74 ± 0.05	47 ± 13	38 ± 8	4869 ± 450	5125.5 ± 441.0	845 ± 13

Table S1. Summary of the surface areas, porosities and cycling characteristics of CNC/MWCNT cathodes.

CNC/MWCNT Property	Fit Parameters							
	Fit	ρ_{CNC}	ρ_{MWCNT}	b	m	A	μ	σ
Surface Area	Linear	200.1	108.9	---	---	---	---	---
Pore Volume	Log-normal + Linear	---	---	0.69	0.04	0.23	-0.75	0.99
Weighted Average Pore Diameter	Gaussian + Linear	---	---	77	-32.6	3.42	0.63	0.22
Gravimetric Discharge Capacity	Log-normal + Linear	---	---	5131.7	-671.9	1061.9	-0.65	0.69
Volumetric Discharge Capacity	Log-normal x Linear	---	---	2691.1	1025.0	2139.5	0.23	0.94
Cycle Lifetime	Gaussian	---	---	44.6	---	11.17	0.49	0.18

Table S2. Parameters determined from data fitting detailed in this manuscript.

CNC Mass Fraction	Cathode Pore Volume (cm^3/g)	Cathode Porosity (%)	Mesopore Volume (cm^3/g)	% Meso-porosity	Macropore Volume (cm^3/g)	% Macro-porosity	Li_2O_2 Volume (cm^3/g cathode)
0	1.98	76	0.69	35	1.29	65	1.93
0.125	1.74	73	0.78	45	0.96	55	2.08
0.25	1.37	65	0.90	66	0.47	34	2.30
0.375	1.41	66	0.91	65	0.49	35	2.44
0.5	1.28	63	0.88	69	0.40	31	2.20
0.65	1.16	59	0.83	72	0.33	28	2.32
0.75	1.09	56	0.80	73	0.29	27	1.63
1	0.95	50	0.75	79	0.20	21	1.76

Table S3. Total porosity, meso- and macroporosity of CNC/MWCNT cathodes as a function of CNC mass fraction, and the volume of the Li_2O_2 formed as the result of fully discharge each formulation.
